# Identification *Mycobacterium* spp. in the Natural Water of Two Austrian Rivers

**DOI:** 10.3390/microorganisms8091305

**Published:** 2020-08-27

**Authors:** Mohammad Reza Delghandi, Karoline Waldner, Mansour El-Matbouli, Simon Menanteau-Ledouble

**Affiliations:** Clinical Division of Fish Medicine, University of Veterinary Medicine, 1210 Vienna, Austria; delghandim@staff.vetmeduni.ac.at (M.R.D.); waldnerk@staff.vetmeduni.ac.at (K.W.); Mansour.El-Matbouli@vetmeduni.ac.at (M.E.-M.)

**Keywords:** molecular epidemiology, nested PCR, environmental mycobacteria

## Abstract

Nontuberculous mycobacteria constitute a subgroup among the *Mycobacterium* genus, a genus of Gram-positive bacteria that includes numerous pathogenic bacteria. In the present study, *Mycobacterium* spp. were detected in natural water samples from two Austrian rivers (Kamp and Wulka) using three different primers and PCR procedures for the identification of the 16S rRNA and *hsp65* genes. Water samples were collected from the Kamp (45 samples) and Wulka (25 samples) in the summer and winter of 2018 and 2019. Molecular evidence showed a high prevalence of *Mycobacterium* sp. in these rivers with prevalence rates estimated at approximately 94.3% across all rivers. The present study represents the first survey into the prevalence of *Mycobacterium* sp. in natural water in Austria. Because nontuberculous mycobacteria have known pathogenic potential, including zoonotic, these findings may have implications for health management and public health.

## 1. Introduction

*Mycobacterium* spp. belong to the family *Mycobacteriaceae* and are Gram-positive, nonmotile, facultative aerobic acid fast bacilli [1]. This genus is found under a wide geographical area, encompassing a wide range of optimal growing temperatures (25–35 °C) [2,3]. Most species of *Mycobacterium* spp. are labelled “nontuberculous mycobacteria” (NTM), a term that excludes the members of the *M. tuberculous* complex and *M. leprae*, as well as a few closely related species, which have historically represented the members of this genus with the most severe impact on human health [4,5]. NTM have been further subdivided between three different groups, based on their virulence and ability to establish an infection. These include true pathogens (*M. marinum*, *M. ulcerans*), opportunistic pathogen (*M. chelonae-abscessus* complex, *M. fortuitum*, *M. avium* complex, *M. haemophilum*, *M. xenopi*, *M. kansassi* and *M. simiae*) and a third group composed of saprophytes mycobacteria (*M. smegmatis*, *M. vaccae*, *M. terrae* complex and *M. gordonae*) [3]. Members of the *Mycobacterium* genus are all considered to be slow-growing. However, there are stark differences between their growth patterns. Consequently, growth kinetic has been used alongside pigmentation patterns as a criterion for the basis of a classification scheme for NTM. While the fast-growing mycobacteria are able to produce colonies visible with the naked eye on solid media within 5 days [6], slow-growers can require much longer. In some extreme cases, such as ovine strains of *Mycobacterium avium* subsp. *mycotuberculosis*, the bacteria can take years to produce visible colonies, but weeks or months are more common durations [7]. Consequently, due to the slow bacterial growth rate and the time required for the development of colonies and turbidity on either solid or liquid media, diagnosis based on bacterial isolation and colony morphology is not considered an appropriate procedure to identify *Mycobacterium* spp. Several other methods have been utilized for the identification of this genus, in particular molecular diagnostic methods based on the DNA or RNA [8].

NTM are found in a wide range of geographical locations and they have been isolated from a variety of samples originating from many different environmental conditions, including low organic matter concentrations and low oxygen level concentrations [9]. These mycobacteria have been reported from water, biofilm and soil and have been found in association with infections in a wide range of hosts, such as mammals, including humans, and birds as well as aquatic animals [3,10,11,12,13]. The most common species identified in water samples include *M. marinum*, *M. gordonae*, *M. flavescens*, *M. fortuitum*, and *M. chelonae* (isolated from aquariums and swimming pools). In addition, other species can cause disease, especially in ornamental fish where *M. triviale*, *M. avium*, *M. abscessus*, and *M. peregrinum* have been regularly associated with diseases [14,15,16], while *M. flavescens* has been more infrequently reported [5]. NTM have a predilection for aquatic environments and it is likely that water plays a significant role as a vector in the transmission of *Mycobacterium* sp. [4]: all of these species have been isolated from several fish species [10,17], and zoonotic cases are often associated with exposure through water or aerosols or handling of contaminated seafood or ornamental fish [18,19,20,21]. Consequently, mycobacteriosis is often linked to the professional occupation of the patients, and people whose occupation involves contact with water and fish are more likely to be exposed to the infection [17,22]. Additionally, due to being frequently reported as a disease from swimmers, the infection has occasionally been referred to as a “Swimming pool granulomas” [17], although this form of the disease is much less common nowadays because of the systematic application of disinfectants. On the other hand, the prevalence of *M. marinum* in natural waters has been estimated at a low level, and it seems that the risk of infection in human is also low [5]. Because of the bacteria’s slow growth and low thermal preferences, infections in humans are often limited to superficial infections with nodules and to the skin and extremities, although deeper infections have also been reported, including deep bursitis, tenosynovitis, arthritis, and osteomyelitis. Moreover, more systemic forms of mycobacteriosis can also occur, including those which involve the respiratory system particularly in immunocompromised patients [5,23]. Additionally, other NTM such as *M. chelonae*, *M. fortuitum*, *M. flavescens*, and *M. gordonae* have also been associated with granulomatous lesions, hepatitis, endocarditis, and meningitis, and infections have been observed in the ocular, bone, joint, and skeletal system [19,21,24].

In fish, *M. avium* has been isolated from Cockatoo Dwarf Cichlid (*Apistogramma cacatuodes*) in the Czech Republic [25]. *M. gordonae* has similarly been reported from several fish species including Gold fish (*Carassius auratus*), Guppy (*Poecilia reticulate*), Angel fish (*Pterophyllum scalare*), and Common carp (*Cyprinus carpio*) [26]. Additionally, *M. fortuitum* and *M. chelonae* have both been reported in the ornamental and wild fish, including Neon tetra (*Paracheirodon innesi*), Goldfish (*Carassius auratus auratus*), Three-spot gourami (*Trichogaster trichopterus*), Cichlid fish (*Microgeophagus altispinosus*), Sterlet (*Acipenser ruthenus*), Siamese fighting fish (*Betta splendens*), Dwarf gourami (*Colisa lalia*), Sailfin molly (*Poecilia latipinna*), Giant sailfin molly (*Poecilia velifera*), Discus fish (*Symphysodon discus*), Green swordtail (*Xiphophorus helleri*), Australian lungfish (*Neoceratodus fosteri*), Silver mullet (*Mugil curema*), Atlantic salmon (*Salmo salar*), and juvenile Pacific salmon (*Oncorhynchus tshawytscha*) [16,24,27,28,29,30,31]. *M. marinum* is an important bacterial agent causing fish tuberculosis, and transmission to humans can be observed via contaminated water in aquarium and fish breeding. It has been associated with salt and fresh water exposure [5]. Furthermore, this species has also been isolated from decorative and farm fish.

Piscine mycobacteriosis is a slow developing chronic disease, although a more acute form of this disease has also been reported, and the disease may not always be associated with obvious clinical signs. Asymptomatic mycobacteriosis has also been reported and is associated with reduced fish growth in aquaculture [17]. When present, clinical signs of mycobacteriosis include nonspecific signs, such as the ones commonly associated with systemic disease in fish such as a swollen abdomen, red lesions on the lateral line, exophthalmia, and pile gills. Additionally, internal signs including organomegaly of the liver, kidney, and spleen have also been reported [6]. A more characteristic sign is the development of granulomatous lesions on the internal organs, which is an uncommon feature in fish. Several virulence factors have been identified in *Mycobacterium* spp. pathogenesis including secretion system 1 (ESX-1) to 5 (ESX-5), PE_PGRs family and PPE proteins (that are considered the most important factor for the replication of *Mycobacterium* sp. in macrophage), and PknG (protein kinase G). The most important virulence factor is the Esx secretion system that is important for both *M. marinum* and *M. tuberculosis* pathogenesis [22,32]. In Austria, the Federal Ministry of Agriculture, Forestry, Environment and Water Management recently announced the objective of increasing national fish production to raise the degree of self-supply from the current 34% to 60%, corresponding to an increase in production from 2400 to 5500 tons annually [33]. This aquaculture production is mostly composed of carp as well as rainbow trout (*Oncorhynchus mykiss*) introduced for farming purposes. Moreover, the endemic brown trout (*Salmo trutta fario*) populates several rivers and waterways. However, these populations are considered at risk as the reported numbers of fish are considered in decline, despite several reintroduction efforts.

Because *Mycobacterium* sp. are known pathogens of wild fish, notably isolated in 2018 from brown trout originating in the Kamp river in Austria [34], we decided to estimate the prevalence of these organisms in the Kamp and the adjacent Wulka river.

## 2. Materials and Methods

### 2.1. Origin of the Water Samples

In total, 70 natural water samples were taken from the Kamp and Wulka rivers on two different sampling dates in 2018 and 2019 as a part of the project ClimateTrout. In total, 45 samples originated from the Kamp and 25 samples originated from the Wulka. The aim of the ClimateTrout project was to investigate the prevalence of the myxozoan *T. bryosalmonae* in wild brown trout and water samples by using PCR, notably in order to determine the role of this parasite in the decline of wild brown trout populations in Austria. The results from this screening were published by Waldner et al. in 2019 [35], and it was decided to use the remaining samples to further investigate additional organisms of interest. Briefly, a 4 L water sample of the Kamp and Wulka rivers was collected and brought to the University of Veterinary Medicine of Vienna. Samples were vacuum filtered with Whatman 1.5 µm GF/F filters (Whatman, Maidstone, United Kingdom) according to Hutchins et al. [36] to concentrate microorganisms. Afterwards, environmental DNA (eDNA) was extracted using the DNeasy Power Soil kit (Qiagen Inc., Hilden, Germany) according to the manufacturer’s instructions. Unfortunately, the water samples did not allow for bacterial isolation by cultures on media.

The samplings took place in June and July 2018 as well as in January 2019 (Table 1), and the water temperature in the rivers at the time ranged from 16 to 22 °C in the summer to 0–2 °C in January.

### 2.2. PCR Assay for the 16S rRNA and hsp65 Genes

Three different PCR procedures and primers sets were used to detect *Mycobacterium* sp. in water samples in order to maximize our confidence in the results. Initially, a PCR assay was performed according to the protocol developed at the University of Veterinary Medicine and published by Delghandi et al. in 2020 [34], using Myco 16F1 (5′-AGCTCGTAGGTGGTTTGTCG-3′) and Myco 16R1 (5′-CCACCTTCCTCCGAGTTGAC-3′) for the detection of the 16S rRNA gene [34]. The total volume of amplification was 25 µL, comprising 12.5 µL Dream Taq Green PCR Master Mix, 1 µL of each primer (10 pmol) and 4 µL eDNA solution. The amplification program consisted of 95 °C for 5 min and 35 cycles of 95 °C for 1 min, 54 °C for 1 min, and 72 °C for 1 min. The resulting PCR amplicon was 611 bp in size.

A second confirmatory nested PCR (nPCR) assay was conducted as previously described by Talaat et al. in 1997 [37] to identify the 16S rRNA gene in members of the *Mycobacterium* genus. Briefly, two different primers were used for the first round and second round PCR (T_39_, T_13_ and T43, T531, respectively). The primers used in the first round amplification were the T_39_ outer F (5′-GCGAACGGGTGAGTAACACG-3′) and T_13_ outer R (5′-TGCACACAGGCCACAAGGGA-3′) primers. Afterward, 2 µL of this product was used in a second round of amplification using the T43 inner F (5′-AATGGGCGCCAAGCCTGATG-3′) and T531 inner R (5′-ACCGCTACACCAGGAAT-3′) primers. The amplification conditions for both rounds were one cycle of 95 °C for 5 min and 30 cycles of 94 °C for 1 min, 50 °C for 1 min, and 72 °C for 1 min. The nPCR assay produced a 300 bp amplification product.

Finally, we also utilized Tb11 (5′-ACCAACGATGGTGTGTCCAT-3′) and Tb12 (5′-CTTGTCGAACCGCATACCCT-3′) to identify a 65 kDa heat shock protein (*hsp65*) gene of *Mycobacterium* according to the procedure described by Telenti et al. [38]. The amplification for these primer pairs was carried out as follow: one cycle of 95 °C for min, followed by 45 cycles of denaturing at 94 °C for 1 min, annealing at 60 °C for 1 min, and extension at 72 °C for 1 min. The resulting amplicon was 439 bp in size.

Each set of samples for the round of amplification included a negative control (using genomic DNA from the Gram-positive aquatic bacterium *R. salmoninarum*) as well as a positive control in the form of DNA extracted from a pure culture of *M. marinum* on Middlebrook 7H10 agar extracted using a DNeasy kit (Qiagen) according to the manufacturer’s instructions. Eight microliters of each PCR product were analyzed by gel electrophoresis on 1% agarose gels and examined under UV illumination.

All samples were screened two times with all three PCR protocols in order to confirm the results. The PCR amplicons were cut from the agarose gels, and DNA were extracted utilizing the MinElute Gel Extraction kit (Qiagen Inc.). Nine positive samples were randomly selected from both rivers; four microliters of each primer (T531, Myco 16F1, and Tb11) in 5 pmol concentration was added to purified samples and sent for sequencing to LGC Genomics Company (Berlin, Germany) by Sanger sequencing to confirm that these samples were homologous to sequences from known members of the *Mycobacterium* genus; sequencing results were analyzed for homology using BLAST (Basic Local Alignment Sequence Tool; National Center for Biotechnology Information; USA). Afterwards, a ClustalW analysis was conducted on the 16s RNA sequences using the software Clustal Omega from the European Bioinformatics Institute of the European Molecular Biology Laboratory (EMBL-EBI). In addition, we added the corresponding sequences from *Mycobacterium* sp. isolated from fish in our previous survey as well as three sequences from known strains of *Mycobacterium* from the NCBI database.

## 3. Results

**Figure 1:**
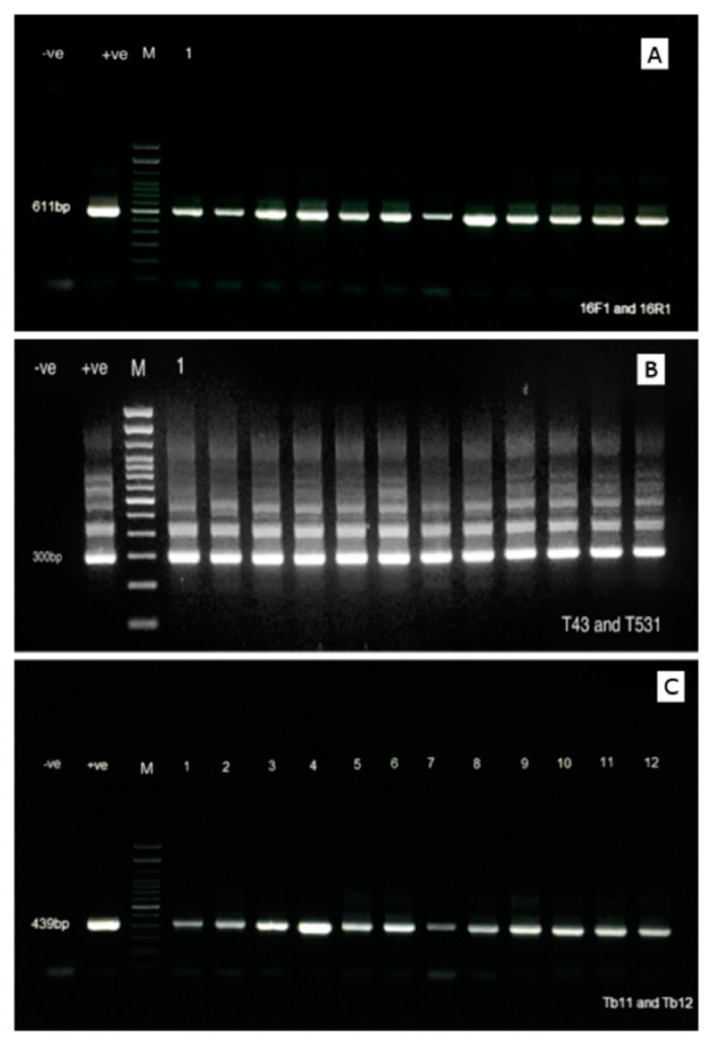
Agarose gel electrophoresis showing the amplicons generated by the various PCR conducted on the positive samples through the 3 different procedures: (**A**) 611 bp amplicon product generated using the PCR procedure described by Delghandi et al. [34]; (**B**) 300 bp amplicon generated using primers targeting the 16S rRNA according to the procedure described by Talaat et al. [37]; (**C**) 439 bp amplicon generated using the primers targeting the *hsp65* gene of *Mycobacterium* sp. according to the procedure described by Telenti et al. [38]. For each gel, 5 µL of the amplicons was loaded in each well.

In total, 45 water samples were collected from the Kamp and 25 samples from the Wulka River. Genomic DNAs were extracted from these samples, and PCRs were performed for each sample in order to detect the presence of NTM based on three different PCR protocols by Delghandi et al. [34], Talaat et al. [37], and Telenti et al. [38] (Figure 1). Notably, the results were identical for all three PCR protocols, and *Mycobacterium* sp. were detected in all samples originating from the Kamp River, at both sampling time points (June and January, Table 1). In the Wulka, the prevalence was also high: all samples collected in the summer 2018 were positive, while only 11 out of 15 samples collected in the winter 2019 were positive (prevalence of 73.33%). There were no significant effect of month or place of sampling (*p* > 0.5). When comparing with previous results regarding the screening of *Mycobacterium* sp. in wild brown trout, all fish that had been found infected with *Mycobacterium* sp. originated from the Kamp River, which had the highest prevalence in the present study [34].

Analysis of the 16S rRNA and *hsp65* sequences confirmed that the bacteria detected most likely belonged to the genus *Mycobacterium*, and the sequences were between 98.5% and 99.2% and 94.2% and 95.0% identical to other sequences from *Mycobacterium* species when sequencing the amplicons generated using the Talaat and Telenti primers, respectively. Similarly, the primers Myco 16 F1 and Myco 16 R1 produced amplicons with 92.8–99.22% identity with the sequences from other mycobacterial species. Notably, none of the three primer-pairs were specific for a single species of *Mycobacterium*, and sequencing always matched more than one species (see Supplementary Table S1). The sequences were deposited in the GenBank database under accession number PRJNA647541.

## 4. Discussion

This survey aimed to investigate the prevalence of *Mycobacterium* sp. in water. *Mycobacterium* spp. are important organisms associated with both aquaculture and human diseases. While members of the genus *Mycobacterium* are considered common inhabitants of aquatic environments, including rivers, lakes, ponds, and streams, there has been no previous study regarding the prevalence of *Mycobacterium* sp. in natural Austrian waters. However, members of the *Mycobacterium* genus have been frequently isolated from water samples as an environmental bacterium. For example, *M. fortuitum* and *M. chelonae* represent the species most frequently isolated from tap water and reservoirs [5]. *M. avium* subsp. *paratuberculosis* has similarly been isolated and identified from the Taff river in Southern Wales using both PCR and culture on Herrold’s egg yolk medium (HEYM) [39] and reported a geographical correlation between the presence of these bacteria and the prevalence of Crohn’s Disease in the population. Notably, culture attempts using *M. avium* have shown that the bacterium was unable to grow when exposed to high NaCl concentrations; on the other hand, its growth rate was enhanced under low concentrations of dissolved oxygen [40]. *M. gordonae* has been frequently isolated from contaminated water [5]; more importantly, this pathogen has been isolated from tap water from hospitals and homes in Germany by Peters et al., using isolation and culture methods [41], which has important public health implications. Moreover, Le Dantec et al. isolated these organisms from membrane filtered water samples originating from the Paris water distribution system on Lowenstein–Jensen medium followed by sequencing of the 16S rDNA gene [42]. NTMs were more common in this study with 78% of the samples being positive for *Mycobacterium* sp. and about 15% contaminated with *mycobacteria* with pathogenic potential [42]. Moreover, Chilima et al. detected *Mycobacterium* sp. using both Ziehl–Nielsen staining and PCR amplification of the 16S rRNA gene in both water and soil samples from Northern Malawi [43]. Notably, these two approaches resulted in very different results with 75% of the samples appearing positive using the staining method, while *Mycobacterium* DNA was only detected in 54% of them [43]. However, the investigators were unable to identify the bacterial isolates at the species level. Concerning fish farms and aquaculture, mycobacteriosis-causing *M. marinum* was observed in rainbow trout and brown trout fish farm population in Italy [44]. Other *Mycobacterium* spp. that were frequently reported in water included *M. kansasii* and *M. xenopi*. While, *M. kansasii* has been rarely reported in aquaculture. This species was isolated from zebrafish (*Danio rerio*) by Kusar et al. in 2017 [45]. However, there is no report of isolation of *M. xenopi* in aquaculture. Additionally, *Mycobacterium* sp. were present in two Finnish lake water samples, and this organism was detected by Niva et al. in 2006 using PCR procedures [46]. Interestingly, *M. pseudoshotsii* has been detected in water in the Chesapeake Bay. This species was isolated frequently in striped bass (*Morone saxatilis*) in this region [47]. In addition, *M. fortuitum* and *M. chelonae* were identified in water samples collected from freshwater rivers, ponds, and brooks in Iran by Rahbar et al. in 2010 using isolation on Lowenstein–Jensen (LJ) medium [48]. Notably, these species have a potential to infect fish (farmed and wild fish) [27,28] and humans [24]. Likewise, *Mycobacterium* spp. have been isolated from tank water and aquariums, and *M. marinum* was reported from aquariums causing infection in humans [49].

Environmental mycobacteria can survive under a wide range of environmental conditions. They have been classified as atypical mycobacteria and are considered opportunistic [50]. Remarkably, all samples were positive with the exception of four samples that had been collected from the river Wulka during the winter. This high level of prevalence of *Mycobacterium* sp. in our samples was consistent with our previous findings, published in 2020 [34], where screening of kidney samples for DNA sequences from *Mycobacterium* sp. in wild brown trout discovered a high prevalence in the Kamp river in June 2018. Interestingly, all of the positive fish samples in this study originated from the same sampling location and time, which could suggest that an outbreak of NTM was taking place in the population at the time of the sampling [34].

**Figure 2:**
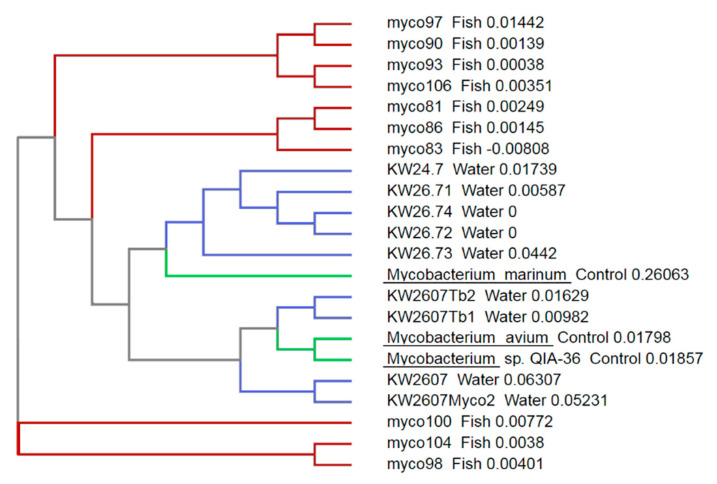
Phylogenetic tree constructed by ClustalW analysis of the 16S rRNA sequences from the amplicons from the present study. Sequences of these amplicons (water, indicated by blue branches) were compared to sequences from fish samples obtained during our previous samples of wild fish in the same rivers (fish, indicated by red branches, see Delghandi et al. [38]) and that of three control *Mycobacterium* sp. from the NCBI dataset (control, indicated by green branches).

When compared with the results from our previous survey performed in wild brown trout [34], the species detected in the present survey appeared more diverse (Figure 2). This could be explained by the fact that the fish sampled in the previous survey originated from a single outbreak, and so it would be plausible that all bacteria involved originated from the clonal expansion of a single bacterial cell, while the present survey involves bacteria from a comparatively large geographic and temporal area. Our samples also bracketed several known mycobacterial species, suggesting that more than one species was detected here; although, because of the lack of specificity at the species level of the primers used in the study, such conclusions are difficult to make.

It is indeed important to note that none of the three sets of protocols and primers were found to be specific at the species level and did not allow for the specific identification of pathogenic mycobacteria. Moreover, molecular methods are also able to detect bacterial DNA even in the absence of biologically active pathogens [51] and as a result do not discriminate between live and dead organisms. Therefore, the actual risk for public health and fish farm associated with this high prevalence is difficult to assess. On the other hand, other investigation projects have made use of isolation and cultures on specific agars. While this approach has the advantage of increased specificity, because it only detects live bacteria and allows for further tests to identify the bacteria at the species level, it is not considered as sensitive, as *Mycobacterium* spp. are difficult to cultivate and are easily outgrown by other environmental bacteria. Another more recently developed technique is immunomagnetic separation polymerase chain reaction (IMS-PCR) where samples are incubated with antibody-coated immunomagnetic beads, to allow the purification of samples. Whan et al. have developed an IMS-PCR method for the detection of *M. avium* subsp. *paratuberculosis* [52] and screened 192 samples of untreated water from Northern Ireland, detecting the bacterium in 15 (8%) of these samples [53].

In the future, it would be beneficial to perform a more thorough investigation, for example, using more diverse sampling, including other rivers and bodies of water as well as various sampling times covering other months and seasons. It would also be beneficial to use a combination of techniques and approaches, in particular, decontamination, for example using NaOH or antibiotics followed by isolation on specific agar and identification of the isolates at the species level, for example using mass spectrophotometry, to maximize the quality of our results. This would allow for a better understanding of the public health risks associated with the presence of *Mycobacterium* sp. in Austrian waters.

## Figures and Tables

**Table 1:** *Mycobacterium* sp. identified in water sample in Austrian rivers.

River Sites	Sampling		Number of Positive/Prevalence Rate of *Mycobacterium* sp.
	Date	Number	
**Kamp**	June 2018	25	25/25 (100%)
	January 2019	20	20/20 (100%)
**Wulka**	July 2018	10	10/10 (100%)
	January 2019	15	11/15 (73.33%)
**Totals**	Total for 2018	35	35/35 (100%)
	Total for 2019	35	31/35 (88.57%)
	Both years	70	66/70 (94.28%)

## Supplementary Materials

The following are available online at https://www.mdpi.com/2076-2607/8/9/1305/s1. Table S1: Most similar sequences for the amplicons in the database of the National Center for Biotechnology Information, based on the results from a search using the Basic Local Alignment Tool.

## Abbreviations

NTM	Nontuberculous mycobacteria
eDNA	Environmental DNA
IMS-PCR	Immunomagnetic separation polymerase chain reaction
nPCR	Nested PCR
BLAST	Basic Local Alignment Sequence Tool
HEYM	Herrold’s egg yolk medium
IMS-PCR	Immunomagnetic separation polymerase chain reaction
PPE proteins	Proline-Proline-Glutamic Acid proteins
