# Supplementary material for: Identification Mycobacterium spp. in the Natural Water of Two Austrian Rivers
Source: Microorganisms. 2020 Aug 27;8(9):1305. doi: 10.3390/microorganisms8091305 (PMC7563569; doi:10.3390/microorganisms8091305)
Supplement: Supplementary file 1 [file microorganisms-08-01305-s001.pdf]

Supplementary Table S1. Most similar sequences for the amplicons in the database of the National Center for Biotechnology Information, based on the results from a search using the Basic Local Alignment Tool. These results only shows the closest match with identified bacteria. Match with sequences belong to unidentified organisms or organisms only identified at the phylum or higher level are omitted.

**Sample name**	Highest matching score among identified bacteria	E-value	Percentage identity
KW24.7	*Mycobacterium* sp. strain R.N1S7 (Accession #: MH298485.1)	$3e^{-127}$	99.22%
KW2607	*Mycobacterium* sp. strain DL189 (Accession #: MH290164.2)	$5\ e^{-84}$	95.43%
KW2607Myco2	*Mycobacterium sp. strain A33* (Accession #: MF102126.1)	0.0	92.84%
KW2607Tb1	*Mycobacterium* sp. LTG 466 (Accession #: KY853653.1)	$3e^{-168}$	95.04%
KW2607Tb2	*Mycobacterium* sp. LTG 466 (Accession #: KY853653.1)	$3e^{-163}$	94.26%
KW26.71	*Mycobacterium* sp. strain AZCC_0280 (Accession #: MK875943.1)	$5\ e^{-125}$	98.47%
KW26.72	Uncultured *Mycobacterium* sp. (Accession #: MG804654.1)	$3e^{-126}$	99.22%
KW26.73	Uncultured *Mycobacterium* sp. (Accession #: MG804654.1)	$4\ e^{-125}$	98.84%
KW26.74	Uncultured *Mycobacterium* sp. (Accession #: MG804654.1)	$3e^{-126}$	99.22%
